# Sternal resection for recurrent breast cancer: a cautionary tale

**DOI:** 10.3747/co.v15i4.226

**Published:** 2008-08

**Authors:** L. Lee, A. Keller, M. Clemons

**Affiliations:** * Division of Medical Oncology, Princess Margaret Hospital, Toronto, ON.; † Department of Radiology, Princess Margaret Hospital, Toronto, ON.

**Keywords:** Breast cancer, sternal metastasis, sternectomy

## Abstract

The occurrence of a solitary sternal metastasis from breast cancer is relatively uncommon, and its treatment is controversial. Most case reports on the role of sternal resection in what is termed a “solitary sternal metastasis” tend to present a rather optimistic outcome.

Here, we report the case of a premenopausal woman with axillary lymph node–positive, triple-negative breast cancer treated with mastectomy followed by adjuvant chemotherapy and radiation therapy. She developed a radiologically isolated sternal recurrence 3 years later, which was treated with partial sternectomy. The present case report reviews the use of sternectomy for breast cancer recurrence and highlights the need for thorough clinical and radiologic evaluation to ensure the absence of other systemic disease before extensive surgery is undertaken.

## 1. CASE REPORT

A 35-year-old woman with a history of breast cancer diagnosed 3 years earlier was referred to us for discussion of bisphosphonate use after surgical excision of an isolated sternal metastasis. Her initial breast cancer was treated with left mastectomy and axillary node dissection. Pathology revealed multifocal, poorly-differentiated invasive ductal carcinoma with three tumour masses (2.3 cm, 2.0 cm, and 1.1 cm). Of 14 lymph nodes, 5 were involved. Margins were negative. Immunohistochemistry was negative for estrogen, progesterone, and human epidermal growth factor (her2/*neu*) receptors. This patient received adjuvant epirubicin/5-fluorouracil/cyclophosphamide (fec-100) chemotherapy for 6 cycles and locoregional radiation treatment. Subsequently, she also had a prophylactic contralateral mastectomy and bilateral transrectus abdominal muscle flap reconstructions. Genetic testing was negative for *BRCA1* and *BRCA2* mutations.

**FIGURE 1:**
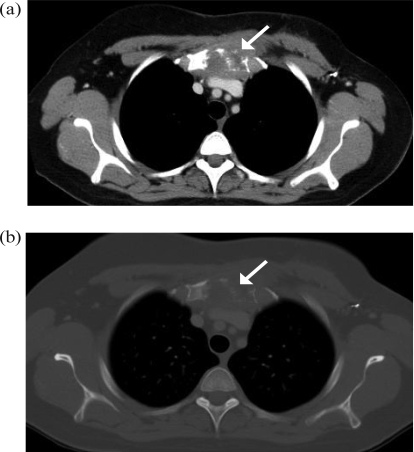
Computed tomography of the thorax, performed preoperatively, shows a soft-tissue mass with manubrial destruction (arrows). (A) Soft-tissue window. (B) Bone window.

Three years after completion of adjuvant systemic therapy, this woman presented to her family physician with new-onset chest and neck tenderness during pregnancy. Investigations were deferred for 6 months until she was postpartum. A computed tomography scan of the neck and chest revealed a 5-cm soft-tissue mass with manubrial destruction consistent with recurrent disease (Figure 1). Restaging revealed no other foci of metastatic disease, and a bone scan showed uptake only in the manubrium. Given the clinical and radiologic impression of an isolated focus of recurrence, the patient elected to have a partial sternectomy.

Pathology revealed a 5.4-cm, poorly differentiated invasive ductal carcinoma infiltrating the manubrium with involvement of the left sternocleidomastoid muscle and left clavicular head. Three lymph nodes were positive (one sternocleidomastoid and two thymic). Margins were clear. Once again, immunohistochemistry was triple-negative.

There were no postoperative complications, and after a 6-week recovery, the woman was referred to our team for a second opinion regarding bisphosphonate use following surgical excision of the sternal metastasis. At presentation, she was asymptomatic. On physical examination, the scars from her breast reconstructions and sternal surgery were evident. She also had two palpable 0.5-cm right supraclavicular nodes.

**FIGURE 2:**
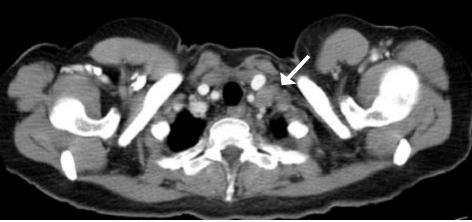
Computed tomography of the thorax, performed postoperatively, shows a 1.6-cm left supraclavicular lymph node deep to the sternocleidomastoid muscle.

Restaging radiologic imaging performed 6 weeks after her surgery confirmed sub-centimetre right supraclavicular lymph nodes and also an enlarged 1.6-cm left supraclavicular lymph node (Figure 2). Fine-needle aspiration of the latter node was positive for triple-negative, poorly differentiated invasive ductal carcinoma.

## 2. DISCUSSION

In patients with breast cancer, the presence of either sternal involvement or an isolated sternal metastasis is relatively uncommon, with reported incidences of 5.2% and 1.9%–2.4% respectively 1,2. Sternal involvement may occur either from direct invasion by enlarged internal mammary lymph nodes or from hematogenous spread. However, in contrast with vertebral lesions, which tend to result in multicentric bony disease from spread through the paravertebral plexus 3, some sternal lesions have been observed to remain solitary with time and may be amenable to surgical resection with curative intent 4,5.

Sternectomy for isolated breast cancer recurrence remains a controversial issue, and the literature consists predominantly of retrospective case series. Noguchi *et al.* 6 performed sternal resections with parasternal and mediastinal lymph node dissection on 9 patients before chemo-endocrine therapy. Eventual relapse in 8 patients revealed that lymph node dissection had no effect on locoregional control. Nevertheless, the dissection provided prognostic information, because all patients with involved parasternal and mediastinal lymph nodes relapsed and died within 30 months, but 3 patients without lymph node involvement survived for more than 6 years.

Lequaglie *et al.* 7 performed radical, curative-intent sternectomies in a subgroup of 28 patients with isolated breast cancer recurrence and found that the 10-year overall survival in the group was 41.8%. These authors suggested that sternectomy could be curative in carefully selected patients.

An isolated sternal metastasis should, however, be regarded with caution, because it is more likely to herald systemic disease than to be truly solitary. Kwai *et al.* 1 demonstrated that 54% of patients with breast cancer and solitary sternal metastasis developed other foci of distant disease within 20 months. The predominance of pulmonary metastasis and distant skeletal disease found is their study was attributed to drainage of the internal mammary nodes into the subclavian vein.

Consequently, patients with breast cancer presenting with a solitary sternal metastasis require thorough restaging evaluation to rule out other foci of metastatic disease. Treatment should be based on a multimodality approach. With an isolated recurrence, local therapy with radiation would be appropriate. In the presence of distant disease, systemic options should be offered. Surgical resection should be reserved for palliation or for instances in which the other treatment modalities are not possible.

Furthermore, the natural history of the initial breast cancer and the prognosis following recurrence should be considered. An important aspect of the present case is that our patient had a triple-negative breast cancer with axillary node involvement. Compared with other breast cancers, this disease subtype is highly aggressive, with an increased rate of early development of distant recurrence that peaks at 3 years after diagnosis 8. Progression is rapid, with a median survival of only 9 months following recurrence. In addition, the extensive literature on relapsed breast cancer demonstrates that a shorter disease-free interval from time of initial treatment is associated with worse prognosis 9.

## 3. CONCLUSIONS

Although resection of a so-called isolated sternal metastasis is surgically feasible, it should be assessed and discussed with patients on a case-by-case basis. In the case of our patient, her history of a treated lymph node–positive tumour with triple-negative phenotype and recurrence within 3 years following mastectomy, chemotherapy, and radiotherapy should realistically have predicted this tragic outcome of widespread relapse within weeks following her extensive palliative surgery.
